# Salivary ATR-FTIR Spectroscopy Coupled with Support Vector Machine Classification for Screening of Type 2 Diabetes Mellitus

**DOI:** 10.3390/diagnostics13081396

**Published:** 2023-04-12

**Authors:** Douglas Carvalho Caixeta, Murillo Guimarães Carneiro, Ricardo Rodrigues, Deborah Cristina Teixeira Alves, Luís Ricardo Goulart, Thúlio Marquez Cunha, Foued Salmen Espindola, Rui Vitorino, Robinson Sabino-Silva

**Affiliations:** 1 Innovation Center in Salivary Diagnostic and Nanotheranostics, Department of Physiology, Institute of Biomedical Sciences, Federal University of Uberlandia, Uberlandia 38408-100, Minas Gerais, Brazil; 2 Faculty of Computing, Federal University of Uberlandia, Uberlandia 38408-100, Minas Gerais, Brazil; 3 Institute of Biotechnology, Federal University of Uberlandia, Uberlandia 38408-100, Minas Gerais, Brazil; 4 School of Medicine, Federal University of Uberlandia (UFU), Uberlandia 38408-100, Minas Gerais, Brazil; 5 Institute of Biomedicine, Department of Medical Sciences, University of Aveiro, 3810-193 Aveiro, Portugal

**Keywords:** diabetes, ATR-FTIR, support vector machine, machine learning, salivary biomarkers, diagnosis, saliva

## Abstract

The blood diagnosis of diabetes mellitus (DM) is highly accurate; however, it is an invasive, high-cost, and painful procedure. In this context, the combination of ATR-FTIR spectroscopy and machine learning techniques in other biological samples has been used as an alternative tool to develop a non-invasive, fast, inexpensive, and label-free diagnostic or screening platform for several diseases, including DM. In this study, we used the ATR-FTIR tool associated with linear discriminant analysis (LDA) and a support vector machine (SVM) classifier in order to identify changes in salivary components to be used as alternative biomarkers for the diagnosis of type 2 DM. The band area values of 2962 cm^−1^, 1641 cm^−1^, and 1073 cm^−1^ were higher in type 2 diabetic patients than in non-diabetic subjects. The best classification of salivary infrared spectra was by SVM, showing a sensitivity of 93.3% (42/45), specificity of 74% (17/23), and accuracy of 87% between non-diabetic subjects and uncontrolled type 2 DM patients. The SHAP features of infrared spectra indicate the main salivary vibrational modes of lipids and proteins that are responsible for discriminating DM patients. In summary, these data highlight the potential of ATR-FTIR platforms coupled with machine learning as a reagent-free, non-invasive, and highly sensitive tool for screening and monitoring diabetic patients.

## 1. Introduction

Diabetes mellitus (DM) is a metabolic disorder characterized by hyperglycemia and glucose intolerance, which are related to defects in insulin secretion and/or the reduced effect of insulin on glucose uptake in peripheral tissues such as skeletal muscle, adipose tissue, and the liver [1,2]. The severity of these comorbidities is strongly related to glycemic control and late diagnosis [3]. This endocrine disorder is a worldwide epidemic and presents a significant rate of morbidity, diabetes-associated mortality, and higher health costs for its management and treatment [4]. According to the International Diabetes Federation (IDF) report of 2019, 1 in 11 adults have DM, a total of 463 million people worldwide, and proximally 232 million people are still undiagnosed [5]. A rapid and early diagnosis of DM becomes imperative to avoid several short-term and long-term complications and improve the health-related quality of life.

The gold standard for diagnosis of DM is blood analysis and this may be diagnosed based on plasma glucose criteria, either the fasting plasma glucose or the 2-h plasma glucose value during a 75 g oral glucose tolerance test (OGTT) or glycated hemoglobin (HbA1c) criteria [6]. The most widely used tool to assess DM progression is the glucose test strips for a point-of-care glucometer, which is currently feasible for screening, monitoring, and diagnosing diabetes by needle finger punctures [7]. However, the constant need of piercing the fingers several times daily is inconvenient, painful, and may lead to the development of finger calluses, which may make blood collection difficult [7,8]. The glucose test strips for a point-of-care glucometer are considered a high-accuracy device to monitor glycemia; however, many patients are reluctant to undergo this procedure several times per day due to its invasiveness and high costs. 

Attenuated total reflection Fourier-transform infrared (ATR-FTIR) spectroscopy is a highly sensitive and reproducible technique capable of characterizing the molecular fingerprint of a sample without extensive sample preparation [9]. It can provide label-free, sustainable, rapid, non-destructive, and cost-effective analyses of multiple components in biological samples [10,11,12,13]. In this context, ATR-FTIR provides an alternative to identify changes in carbohydrates, proteins, lipids, DNA, and RNA promoted by diseases, which can indicate spectral infrared biomarkers that correlate with gold-standard clinical biomarkers [9,14]. Minimally or non-invasive screening or diagnostic tests facilitate compliance and, as such, saliva collection is an attractive sampling method. An infrared diagnostic system for diabetes using saliva with linear discriminant analysis (LDA) showed an accuracy of 88.2%. This protocol was used with a higher amount of sample dried with continuous airflow and the saliva was inserted in barium fluoride (BaF2) windows, which limits sequential analysis due to 30 min drying for each sample and use of a potentially toxic element [15]. In addition, the salivary monitoring of DM was successfully determined using the FTIR analyses along with the PCA method, with 95.2% accuracy in rat samples [16]. 

Here, we hypothesized that ATR-FTIR is capable of identifying changes in salivary components to be used as a screening tool for DM. The present study aimed to compare infrared salivary components in normoglycemic non-diabetic subjects and hyperglycemic type 2 diabetic patients to be employed as an alternative salivary biomarker tool for DM screening.

## 2. Materials and Methods

### 2.1. Ethical Aspects and Study Subjects

The study was conducted at the Clinics’ Hospital of the Federal University of Uberlandia (HC-UFU, Uberlandia, Minas Gerais, Brazil). All experimental procedures were carried out in accordance with the Code of Ethics of the World Medical Association (Declaration of Helsinki) and were approved by the Institutional Review Board of the Federal University of Uberlandia (protocol number 1.715.975). Written informed consent was obtained from all the participants of this study, including controls and patients. The research protocol also followed the Standard for Reporting of Diagnostic Accuracy Studies (STARD) statement.

The inclusion criteria were: adults between 18 and 85 years of age, both genders, patients from the Endocrinology Outpatient Clinic of the Clinic Hospital, Federal University of Uberlandia (HC-UFU), diagnosed with type 2 diabetes mellitus in any moment or patients without diabetes mellitus, who agree to participate in the research, thus signing the written informed consent. Patients with a recent history of surgery, trauma, cancer, autoimmune diseases, transplants, patients with orthodontic treatment, other dental treatment, pregnancy or lactation, and any other specific condition that may interfere with the results of the biochemical pattern of saliva were not included in the study. The attendance of patients in hospital is related to a high level of stress and ATR-FTIR spectroscopy can detect physiological stress in salivary composition. The sample in non-diabetic subjects was defined for convenience; we collected samples in exactly the same conditions for both groups to block the effect of physiological stress in our study.

The study group included 68 subjects: 23 non-diabetic individuals and 45 uncontrolled type 2 diabetic patients. Non-diabetic subjects with glycemia <100 were considered normoglycemic, and diabetics with glycemia >126 mg/dL were considered uncontrolled type 2 diabetic patients with poor glycemic control. This classification is according to the American Diabetes Association (ADA) [6], World Health Organization (WHO) [17], and Diabetes Brazilian Society (SBD) [18]. 

### 2.2. Saliva Sample Collection and Preparation

Saliva samples were collected in Salivette^®^ tubes (Sarstedt, Germany), consisting of a neutral cotton swab and a conical tube. The patient moved the swab for three minutes throughout the oral cavity to collect homogeneous saliva, which was then returned to the tube that was covered with a lid. Saliva was collected before blood collection for routine blood biochemical analyzes at HC-UFU. Then, saliva was recovered by centrifugation for 3000 rpm at 4 °C for 3 min, and the supernatant was collected and aliquoted. All samples were kept frozen at −80 °C until analysis. Saliva collection was standardized by the routine of our study group and adapted from the Compliance with Saliva Collection Protocol [19]. The blood collection was performed immediately after saliva collection, and glycemia and HbA1C values information were obtained by medical records.

### 2.3. ATR-FTIR Spectroscopy

The salivary spectra were performed in 4000–400 cm^−1^ in ATR-FTR spectrophotometer Vertex 70 (Bruker Optics, Reinstetten, Germany) coupled to attenuated total reflectance (ATR) component. The crystal material in ATR unit was a diamond disc as an internal reflection element. The salivary pellicle penetration depth ranges between 0.1 and 2 μm and depends on the wavelength, incidence angle of the beam, and the refractive index of ATR-crystal material. Saliva (1 μL) was directly dried at room temperature on ATR-crystal for 6 min and then the spectra were recorded. Before each sample analysis, the air spectrum was used as a background. Each spectrum was obtained in a room with a temperature around 22–23 °C, 4 cm^−1^ of resolution, and 32 scans were performed [16].

### 2.4. Spectral Data Processing and Statistical Analysis

The spectra data obtained were processed using Opus software, version 6.5 (Bruker Optics, Reinstetten, Germany). Measurements were performed in the mid-infrared region (4000–400 cm^−1^). For the generation of average spectra and band areas, the spectra were vector normalized and baseline corrected by the Rubberband method to avoid errors during sample preparation and spectra analysis. Band positions were measured using the frequency corresponding to the weight center of each band. Band areas were calculated from normalized and baseline-corrected spectra using OPUS software [16].

The Kolmogorov–Smirnov test was applied to test the normality of the variables. The band area data were analyzed using the Student’s *t*-test. Sensitivity and specificity values were calculated from the dataset by applying the ROC curve (Receiver operating characteristic) analysis. All these analyzes were performed using GraphPad Prism software (GraphPad Prism version 7.00 for Windows, GraphPad Software, San Diego, CA, USA). Only *p* values < 0.05 were considered significant and results were expressed as mean ± S.D.

### 2.5. Discrimination Analysis Method

The infrared spectral data analysis was divided into two stages: pre-processing and classification. Pre-processing consisted of aggregation, attribute selection, and data transformation. The arithmetic mean of the three spectral readings of each patient was performed in aggregation. The spectral data were truncated with the lipidic region (3050–2800 cm^−1^) associated with the biofingerprint region (1800–900 cm^−1^). Then, a Savitzky–Golay smoothing filter was applied to each spectrum, followed by a first-order derivative and pre-processed by amide I normalization.

**Figure 1:**
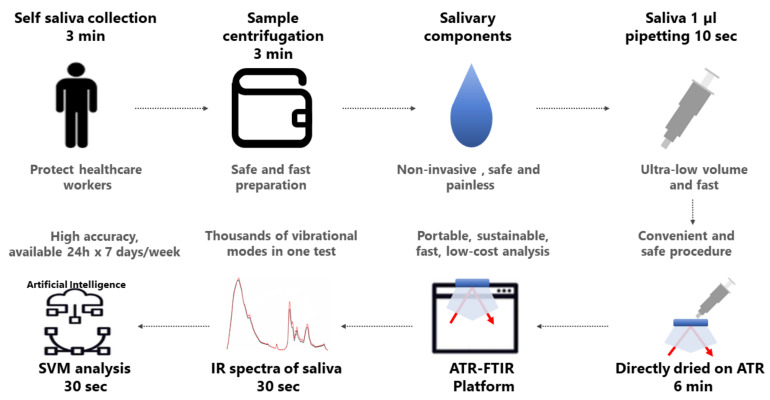
Biophotonic platform for salivary diagnostics of diabetes: proposed workflow for salivary collection and sample preparation; saliva (1 μL) directly dried on ATR-crystal, infrared acquisition on ATR-FTIR platform and discrimination with machine learning algorithms.

The classification was tested with state-of-the-art machine learning algorithms and linear discriminant analysis of feature extraction coupled with discriminant analysis tools. The Linear Discriminant Analysis (LDA) and Support Vector Machine (SVM) were selected based on better results during model training. To analyze the predictive performance of the LDA and SVM algorithms, ten times stratified cross-validation was used. The samples were divided into ten subsets with each iteration; nine of them were used to train the algorithm and one exclusively to test it, so that each subset was part of the test once. In addition, the procedure was repeated three times with changes in the sample configurations in these subsets to achieve a closer estimate of the real performance of the model, thus totaling thirty executions. To measure the results obtained, three performance measures consolidated in the literature were used: sensitivity, specificity, and accuracy. The sensitivity or true positive rate is the proportion of positives (diabetic) that were correctly classified, and the specificity or true negative rate is the proportion of negatives (normoglycemic) that were correctly classified. The accuracy is defined as the total number of samples correctly classified, considering true and false negatives [16,20]. A proposed workflow for a portable, sustainable, rapid, and non-invasive diagnosis platform for diabetes using saliva is shown below (Figure 1).

## 3. Results

### 3.1. Study Subject Characterization 

Demographic and biochemistry data of non-diabetic and diabetic patients are described in Table 1. Briefly, the body weight was higher (*p* < 0.05) in uncontrolled type 2 diabetic patients than in non-diabetic subjects. As expected, glycemia and HbA1C were also higher in uncontrolled type 2 diabetic patients than in non-diabetic subjects. The mean blood glucose and HbA1C value of non-diabetic patients was 98.8 ± 6.7 and 5.2 ± 0.1, respectively, while that of uncontrolled type 2 diabetic patients was 187.0 ± 90.2 and 8.3 ± 1.7, respectively.

### 3.2. FTIR Analysis of Saliva Spectra between Non-Diabetic Subjects and Uncontrolled Type 2 Diabetic Patients

**Figure 2:**
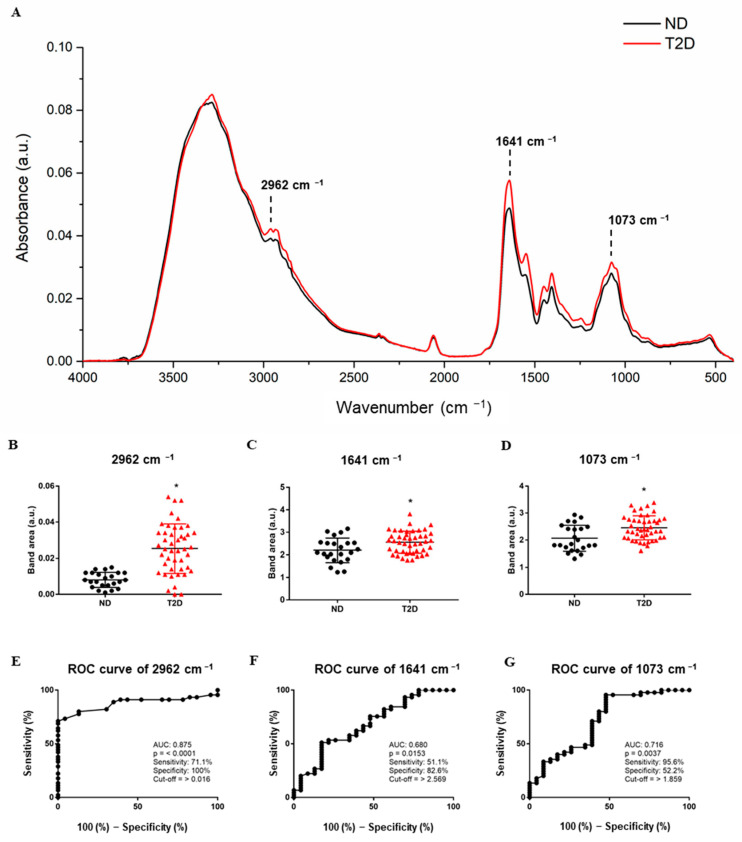
Representative average ATR-FTIR spectra (4000–400 cm^−1^) in saliva of non-diabetic subjects (ND) and uncontrolled type 2 diabetic patients (T2D) (**A**). Band area of 2962 cm^−1^ (**B**), 1641 cm^−1^ (**C**), and 1073 cm^−1^ (**D**). ROC curve analyses of 2962 cm^−1^ (**E**), 1641 cm^−1^ (**F**), and 1073 cm^−1^ (**G**). * Significantly different compared to ND.

As shown in Figure 2A, the infrared salivary spectra of non-diabetic subjects and uncontrolled type 2 diabetic patients contain different molecules such as lipids, proteins, carbohydrates, and nucleic acids. Some bands of interest are shown in Figure 2B–D, which contain band area values of 2962 cm^−1^ (CH3 of lipids), 1641 cm^−1^ (amide I), and 1073 cm^−1^ (carbohydrates and glycosylated proteins) that are increased in uncontrolled type 2 diabetic patients compared to non-diabetic subjects.

Considering that sensitivity and specificity are basic characteristics to determine the accuracy of diagnostic and monitoring tests, ROC curve analysis was used to evaluate the potential diagnostic of these spectral bands. In ROC analysis, the area under the curve (AUC) of 2962 cm^−1^ was 0.875, with a sensitivity of 71.1%, specificity of 100% and the cutoff value was 0.016 (Figure 2E). The band 1641 cm^−1^ showed an AUC of 0.680, a sensitivity of 51.1%, and specificity of 82.6% with a cutoff value of 2.569 (Figure 2F). In relation to band 1073 cm^−1^, the ROC curve analysis demonstrated an AUC of 0.716, with a sensitivity of 95.6%, specificity of 52.2%, and a cutoff value of 1.859 (Figure 2G). These findings indicate that the ROC curve differentiates non-diabetic subjects and uncontrolled type 2 diabetic patients.

### 3.3. Discrimination Analysis

In order to classify and discriminate salivary spectral samples from non-diabetic subjects and uncontrolled type 2 diabetic patients more quickly and with greater reliability, artificial intelligence tools were applied, most notably machine learning with linear discriminant analysis (LDA) and support vector machine (SVM). The classification of salivary infrared spectra by LDA and SVM is described in Table 2. The results obtained in these analyses indicate that the best discrimination was the SVM algorithm with pre-processing using the Savitzky–Golay smoothing filter, followed by a 1st derivative. The best result was obtained using the 3050–2800 cm^−1^ along with 1800–900 cm^−1^. The classification of salivary infrared spectra by the SVM algorithm showed a sensitivity of 93.3% (42/45), specificity of 74% (17/23), and accuracy of 87% between non-diabetic subjects and uncontrolled type 2 diabetic patients.

Shapley Additive Explanations (SHAP) is considered a popular state-of-the-art approach to explaining machine learning models. As a result, SHAP calculates the contribution of each feature to the target value and the SHAP method can be used to analyze the prediction for both classification and regression models [21]. The SHAP value of each feature within the SVM model helps to select which feature is important and which bands are responsible for discrimination by the best algorithm between non-diabetic individuals and uncontrolled type 2 diabetic patients.

**Figure 3:**
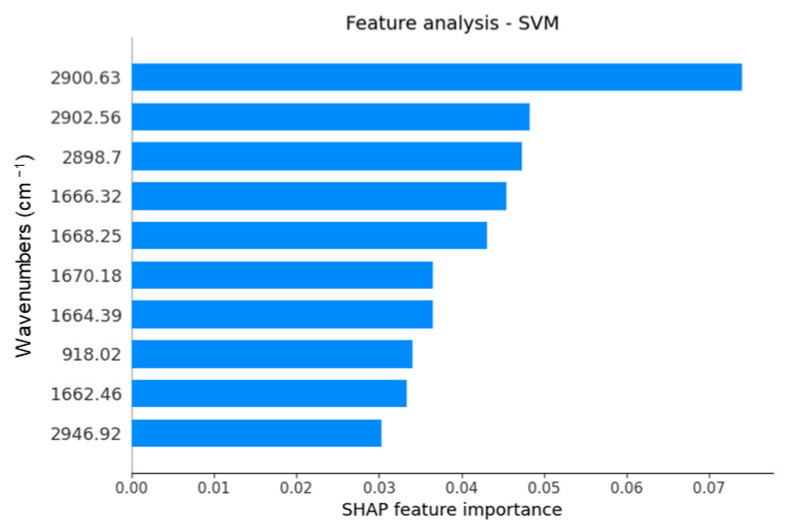
The feature importance (SHAP value) graph of SVM model.

As an outcome, the SHAP feature importance indicates the main salivary vibrational modes at 2900 cm^−1^, 2902 cm^−1^, 2898 cm^−1^, 1666 cm^−1^, 1668 cm^−1^, 1670 cm^−1^, 1664 cm^−1^, 918 cm^−1^, 1662 cm^−1^, and 2946 cm^−1^ as responsible for distinguishing non-diabetic individuals and uncontrolled type 2 diabetic patients (Figure 3). The molecular assignments of each vibrational mode indicated by the SHAP feature analysis—SVM are described in Table 3 [22].

## 4. Discussion

The early diagnosis and adequate monitoring of diabetes can reduce the complications promoted by unappropriated hyperglycemia, which is critical to improving quality of life, saving health care costs, reducing costs related to reduced work productivity and inability to work, as well as early mortality [23,24]. With the direction and progress of the application of portable ATR-FTIR devices in clinical practice [9,25], the development of non-invasive and sustainable reagent-free platforms to detect changes in salivary components of the type 2 diabetic population has great potential to be applied at the point-of-care, at home, or in decentralized laboratorial settings with reduced infrastructure [16].

The levels of glycemia and HbA1C have been considered effective clinical biomarkers for diagnosis and monitoring. These glucose monitoring strategies in the blood are the most widely used and provide an effective method for diabetes surveillance. However, the inconvenience of blood collection can reduce the continuous monitoring of metabolic control and reduce the detection of hyperglycemia intervals [26]. In this context, the ATR-FTIR platform using alternative non-invasive specimens can be more amenable for clinical applications.

The discriminatory vibrational modes in the saliva of hyperglycemic type 2 diabetic patients compared to non-diabetic subjects were related to the asymmetric stretching vibration of CH3 of lipids (2962 cm^−1^), amide I (1641 cm^−1^) and carbohydrates and glycosylated proteins (1073 cm^−1^) [16,27]. In fact, we previously showed that the hypoinsulinemic animal model of diabetes showed changes in lipid, amide I, and asymmetric CH3 bending modes of proteins [16]. The increase in the amide I and carbohydrates/protein glycosylation are in agreement with changes in protein and glucose concentrations in the saliva of diabetes subjects [15,28,29]. However, these findings focused on pointing out significant and unitary changes by univariate analysis, and may have a limitation in relation to reproducibility when applied on a larger scale in clinical practice.

These three salivary spectral modes (2962 cm^−1^, 1641 cm^−1^, and 1073 cm^−1^) showed good sensitivity and specificity in the ROC analysis, with emphasis on the vibrational band 1073 cm^−1^ with 95.6% sensitivity and the peak 2962 cm^−1^ with 100% specificity. These results indicate that these spectral modes can be used as a platform for the diagnosis and screening of DM. Our ROC analysis results were similar to the recent study in which 92% of the ROC curve was indicated using weighted KNN (k-nearest neighbors) as the classifier; however, two healthy individuals and nine DM subjects were used in this pilot study [30].

To improve discrimination and increase the understanding of classification so that it has greater applicability in clinical practice, we used regions of the spectrum (3050–2800 cm^−1^ along with 1800–900 cm^−1^) in a classification process by machine learning algorithms. From the point of view of the diagnostic or screening application, the sensitivity of 93.3% by the SVM algorithm with adequate specificity represents the significant translational potential of this salivary platform to diabetes. Population-based diabetes screening could be an attractive intervention as a result of its prevalence, high medical and non-medical costs and, especially, for the long asymptomatic phase prior to diabetic symptoms. However, the test cost affects the evaluation of cost-effectiveness. From a sustainability perspective, other issues in large population screening programs are the potential toxicity of the reagents and generation of healthcare waste [31,32]. For example, in the ATR-FTIR analysis of blood, it is frequently necessary to insert anticoagulants to avoid blood clotting [33]. The development and clinical assessment of a low-cost, sustainable, reagent-free, non-invasive, and highly sensitive infrared platform using only 1µL of saliva and minimal sample preparation could be a shift paradigm to detect undiagnosed diabetes and increase the chance to treat diabetes early.

The SHAP feature analysis—SVM indicated some vibrational bands that contributed to discriminating between non-diabetic individuals and uncontrolled type 2 diabetic patients. Among the main altered biomolecular components were lipids, protein, and genetic material (DNA). These findings are consistent with the pathophysiology of DM, with increased lipids due to increased lipolysis in adipocytes due to deficient insulinization, resulting in an increased release of fatty acids from adipocytes [34]. Additionally, studies have shown that diabetics have increased inflammatory biosynthesis which contributes to the progression of diabetes [35]. Although the complex cascade of events that lead to cellular dysfunction in response to high glucose levels is not fully understood, one such event is the formation of advanced glycation end products (AGEs), which are lipids and proteins modified by increased blood glucose, capable of altering metabolic functions and increasing inflammation [34,36]. Free radicals derived from glycation can cause protein fragmentation and oxidation of lipids and the amino groups of the adenine and guanine bases in DNA are also susceptible to glycation and AGE formation [36]. This imbalance in metabolic homeostasis creates several chemical compounds that can activate intracellular signaling pathways and produce pro-inflammatory cytokines that lead to the progression of diabetic complications [34,37]. Therefore, the possibility of reducing these complications with a faster, more comfortable, and less invasive monitoring and diagnosis alternative by ATR-FTIR spectroscopy for diabetic patients is necessary.

Invasive diagnosis and monitoring facilitate adherence by the population and, as such, saliva collection is an attractive sample. Thus, an infrared spectroscopy diagnostic system for diabetes using saliva with LDA analysis with an accuracy of 88.2% has already been described [15]. However, in this study, a large volume of salivary sample was used, with a 30-min drying time for each sample and the use of a potentially toxic element in the preparation of the samples [15]. This accuracy result was similar to our findings of 87% using SVM analysis.

A recent study with a large number of participants evaluated unstimulated saliva as a potential biofluid in patients with controlled and poorly controlled diabetes using Artificial Neural Network (ANN), SVM, and Linear Regression (LR) algorithms [38]. In this study, in addition to using a larger volume of sample and longer drying time, a non-diabetic group was not used in its experimental design, which is one of the major limitations. Furthermore, in this study, the spectral regions 4000–400 cm^−1^, 4000–2000 cm^−1^, and 1800–800 cm^−1^ were used for the analysis with the ANN, SVM, and LR algorithms, with the best result being in the region of 4000–2000 cm^−1^ by ANN algorithm [38]. Here, in the present study, in addition to using a methodology with lower sample volume and drying time, we evaluated a different spectral region (3050–2800 cm^−1^ along with 1800–900 cm^−1^), evaluating the entire spectral part of biological interest. In addition, in our study, the objective was to identify changes in salivary components to be used as a screening tool for DM, different from the proposal of Sánchez-Brito et al. (2021), which focused on characterizing controlled and uncontrolled diabetic patients, grouping patients into groups with different glycemic values.

Our exploratory clinical study supports the significant potential towards a salivary screening platform for type 2 diabetes. Despite this recent biotechnological advance for diabetes screening, the predictive accuracy with respect to sensitivity and specificity should be further validated in large clinical trials [39]. Besides, novel studies are also needed to evaluate operational challenges such as access to ATR-FTIR devices and their impact on medical decision making, which is outside the scope of the present study [40]. In this context, the salivary ATR-FTIR spectroscopy coupled with SVM classification could provide a novel alternative for biomedical screening or monitoring. This retrospective patient cohort was not designed per se to test the discrimination between subjects with higher body weight or obese subjects compared to type 2 diabetic patients, as it will be tested in future trials including an analysis with similar body mass index (BMI). Besides, it is important to investigate the impact of comorbidities that often occur with type 2 diabetes, such as hypertension. We can also improve the proposed workflow for salivary collection; salivary preparation to be dried on ATR-crystal, infrared acquisition on the ATR-FTIR platform and discrimination with machine learning algorithms, which could provide more robust results [9,41]. Additional studies are also needed to evaluate the high-throughput biophotonic platform and scalability issues [41,42] in the context of diabetes. Although the macroscale ATR-FTIR can be used in biomedical settings for the diabetes population, the development of lab-on-chip ATR-FTIR devices can be applied in point-of-care (PoC) diagnostics and vastly increase the scalability of biophotonic analysis [43,44], especially for diabetes condition [44]. A lab-on-chip ATR-FTIR device was proposed integrating a microfluidic approach with a pseudo-continuous flow FTIR system for glucose detection [44]. Other miniaturized devices were used to detect solute levels in solution using microchannels [45]. Another recently developed technology is a portable haemoprocessor combined with ATR-FTIR. It was efficiently tested and can be applied in a low-volume of biofluids [43].

## 5. Conclusions

Herein, the present study seeks to contribute to the application of ATR-FTIR platforms in medical and dentistry settings. Here, data indicate 2962 cm^−1^, 1641 cm^−1^, and 1073 cm^−1^ as discriminatory vibrational modes of type 2 diabetes than normoglycemic controls in univariate analysis. The classification of salivary infrared spectra showed a sensitivity of 93.3% (42/45), specificity of 74% (17/23), and accuracy of 87% between non-diabetic subjects and uncontrolled type 2 diabetic patients using SVM analysis. In summary, these data highlight the potential of ATR-FTIR platforms coupled with machine learning as a sustainable, reagent-free, non-invasive, and highly sensitive tool for screening and monitoring diabetic patients.

The data presented here represent a valuable improvement towards clinical applications of biophotonic platforms using non-invasive samples for diabetes detection. Even screening employment using this platform can be suitable for private and public health systems due to several advantages of salivary diagnostics. Thus, this is a promising clinical study for the application of an ATR-FTIR platform coupled with artificial intelligence algorithms for diabetes detection to promote sustainable development.

## Figures and Tables

**Table 1:** Characterization of patients: gender, body weight, age, glycemia, and glycated hemoglobin (HbA1C).

Parameters	Non-Diabetic	Type 2 Diabetes Mellitus
Gender (male rate)	43.4%	57.7%
Body weight (kg)	68.4 ± 8.4	87.17 ± 11.9 *
Age (years)	57.0 ± 10.7	61.9 ± 10.1
Glycemia (mg/dL)	98.8 ± 6.7	187.0 ± 90.2 *
HbA1C (%)	5.2 ± 0.1	8.3 ± 1.7 *

Values are expressed as mean ± S.D; * Significantly different compared to non-diabetic.

**Table 2:** Machine learning algorithms applied in salivary spectra to discriminate non-diabetic individuals and uncontrolled type 2 diabetic patients.

Pre-Processing (Band)	Algorithm	Accuracy	Sensitivity	Specificity
Raw data (1800–900 cm^−1^; 3050–2800 cm^−1^)	Linear Discriminant Analysis	0.71	0.73	0.65
	Support Vector Machine	0.82	0.89	0.70
Rubberband + amida I (1800–900 cm^−1^; 3050–2800 cm^−1^)	Linear Discriminant Analysis	0.54	0.58	0.48
	Support Vector Machine	0.79	0.87	0.65
1st deriv, Savgolay (1800–900 cm^−1^; 3050–2800 cm^−1^)	Linear Discriminant Analysis	0.66	0.73	0.52
	Support Vector Machine	**0.87**	**0.93**	**0.74**

**Table 3:** Band assignment of SHAP value of each feature within the SVM model to discriminate non-diabetic individuals and uncontrolled type 2 diabetic patients and its molecular assignments.

Wavenumber	Band Assignment	Biomolecular Components
2900 cm^−1^	Stretching vibrations of CH_2_ and CH_3_	Phospholipids
2902 cm^−1^	CH_3_ symmetric stretch	Lipids
2898 cm^−1^	CH_3_ symmetric stretch	Lipids
1666 cm^−1^	C=O stretching vibration	Pyrimidine base
1668 cm^−1^	C=O stretching of Amide I	Protein
1670 cm^−1^	Amide I (anti-parallel β-sheet)	Protein
1664 cm^−1^	Amide I	Protein
918 cm^−1^	Left-handed helix DNA (Z form)	DNA
1662 cm^−1^	Amide I	Protein
2946 cm^−1^	Stretching C-H	Lipids

## Data Availability

Not applicable.
